# Case Report: Rotavirus Vaccination and Severe Combined Immunodeficiency in Japan

**DOI:** 10.3389/fimmu.2022.786375

**Published:** 2022-02-23

**Authors:** Kay Tanita, Yoshiki Kawamura, Hiroki Miura, Noriko Mitsuiki, Takahiro Tomoda, Kento Inoue, Akihiro Iguchi, Masafumi Yamada, Taro Yoshida, Hideki Muramatsu, Norimasa Tada, Toshihiro Matsui, Motohiro Kato, Katsuhide Eguchi, Masataka Ishimura, Shouichi Ohga, Kohsuke Imai, Tomohiro Morio, Tetsushi Yoshikawa, Hirokazu Kanegane

**Affiliations:** ^1^Department of Pediatrics and Developmental Biology, Graduate School of Medical Sciences, Tokyo Medical and Dental University (TMDU), Tokyo, Japan; ^2^Department of Pediatrics, Fujita Health University School of Medicine, Toyoake, Japan; ^3^Department of Pediatrics, Faculty of Medicine and Graduate School of Medicine, Hokkaido University, Sapporo, Japan; ^4^Children’s Cancer Center, National Center for Child Health and Development, Tokyo, Japan; ^5^Department of Pediatrics, Nagoya University Graduate School of Medicine, Nagoya, Japan; ^6^Department of Pediatrics, Tsuchiura Kyodo General Hospital, Ibaraki, Japan; ^7^Department of Pediatrics, Graduate School of Medical Sciences, Kyushu University, Fukuoka, Japan; ^8^Department of Community Pediatrics, Perinatal and Maternal Medicine, Graduate School of Medical and Dental Sciences, Tokyo Medical and Dental University (TMDU), Tokyo, Japan; ^9^Department of Child Health and Development, Graduate School of Medical and Dental Sciences, Tokyo Medical and Dental University (TMDU), Tokyo, Japan

**Keywords:** severe combined immunodeficiency, hematopoietic cell transplantation, rotavirus, vaccination, T-cell receptor excision circles (TREC)

## Abstract

Severe combined immunodeficiency (SCID) is an inborn error of immunity that occurs in approximately 1 in 50,000 births, mainly due to impaired lymphocyte differentiation. Without curative treatment, such as hematopoietic cell transplantation (HCT) or gene therapy, severe infection in the first year of life could make this condition fatal. The results of HCT are poor when patients have active infections, thus requiring early diagnosis before onset of infection. In five cases of SCID diagnosed in Japan, the oral rotavirus vaccine had been administered before diagnosis. In this study, we demonstrated that the rotavirus from their stools was a vaccine-derived strain. In some cases, severe gastroenteritis triggered the diagnosis of SCID. However, newborn screening for SCID is available before the first rotavirus vaccination using assays for the detection of T-cell receptor excision circles (TRECs). Therefore, to improve the prognosis of patients with SCID in Japan, we should establish a screening system of TRECs for newborns throughout Japan.

## Introduction

Severe combined immunodeficiency (SCID) is a critical inborn error of immunity (IEI) that causes cellular and humoral immunity failure due to impaired lymphocyte differentiation, resulting in severe infections from infancy. Therefore, hematopoietic cell transplantation (HCT) or gene therapy is required as a curative therapy.

Rotavirus infection is a gastrointestinal infection that occurs in infants and children. Although mild cases can resolve spontaneously, some cases can cause fatal dehydration and encephalitis or encephalopathy, leading to the hospitalization of many patients. Although live vaccination is contraindicated (1), patients with SCID often remain asymptomatic until early infancy and are rarely diagnosed before 2 months of age when oral rotavirus vaccination is initiated. Furthermore, Rotarix^®^, a live, monovalent, attenuated, human rotavirus vaccine (RV1), and RotaTeq^®^, a live, pentavalent, human–bovine reassortant rotavirus vaccine (RV5), were initiated as arbitrary vaccination in 2011 and 2012, respectively, in Japan. Regular administration of these vaccines was initiated in October 2020. This initiative was taken after realizing that the number of cases vaccinated before the diagnosis of SCID will increase.

Several severe cases of rotavirus gastroenteritis caused by vaccine strains in SCID have been reported overseas (2, 3) as well as in Japan (4, 5). This report summarizes the cases of five patients with SCID who were vaccinated before being diagnosed with SCID in Japan.

## Materials and Methods

### RNA Extraction from Patient Stool and Serum

Ten percent suspensions (1 ml) of each stool sample were prepared in physiological saline solution or swab samples were rinsed in 500 μl of physiological saline solution. Each suspension was then centrifuged for 20 min at 4,000 × *g* and 140 μl of the supernatant was used for RNA extraction. Finally, viral double‐stranded RNAs (dsRNAs) were extracted from the stool suspension and 140-μl sera using a QIAamp Viral RNA Mini Kit (Qiagen, Hilden, Germany).

### Sanger Sequencing

First, RNA was extracted from patients’ stool samples and RotaTeq and complementary DNA was synthesized using the High-Capacity cDNA Reverse Transcription Kit (Foster City, CA, USA). DNA was amplified by polymerase chain reaction (PCR) using primers for human rotavirus A gene 10, which encodes the rotavirus *Non-structural protein 4* (*NSP4*) gene, and sequenced using the Sanger method (forward primer: 5’-GGGCTTTTAAAAGTTCTGTTCCGAG-3’, reverse primer: 5’-GGTCACACTAAGACCATTCC-3’). Finally, we compared nucleotide and amino acid sequences of rotavirus strains derived from patients’ stool samples, the RotaTeq WC3 strain, and the wild-type human/Wa strain.

### RNA Extraction and RV1- and RV5-Specific Real-Time Reverse Transcription-Polymerase Chain Reaction

The stool samples were analyzed by real-time reverse transcriptase-polymerase chain reaction (RT-PCR) for the presence of RV5, RV1, and wild-type strains (6), and details about the real-time RT-PCR analysis were described elsewhere (7). Real-time RT-PCR was performed on a Fast Optical 48-Well Reaction Plate using a TaqMan RNA-to-Ct 1-Step kit (Thermo Fisher Scientific, Waltham, MA). In addition, single-well denaturation, reverse transcription, and amplification were performed on a StepOne Real-Time PCR system in standard mode (Thermo Fisher Scientific). Thermocycling conditions included a 15-min hold at 48°C, a 10-min cycle at 95°C, and 45 cycles of 15 s at 95°C and 1 min at 60°C. Each rotavirus vaccine strain-specific real-time RT-PCR amplified the designated vaccine strain only, and no cross-reaction was observed with any of the wild-type strains. Meanwhile, real-time RT-PCR to detect *Non-structural protein 3* (*NSP3*) gene in wild-type strains could also detect the two vaccine strains. Serially diluted purified rotavirus virions were used to determine the lower detection limits of RV5 (10 copies/reaction), RV1 (50 copies/reaction), and wild type (50 copies/reaction) using real-time RT-PCR. While the RNA extracted from RV5 and RV1 was used as a positive control for vaccine virus strains, the KU strain (G1P [8]) was used as a positive control for the wild-type virus.

## Results

### Case Reports

**Figure 1:**
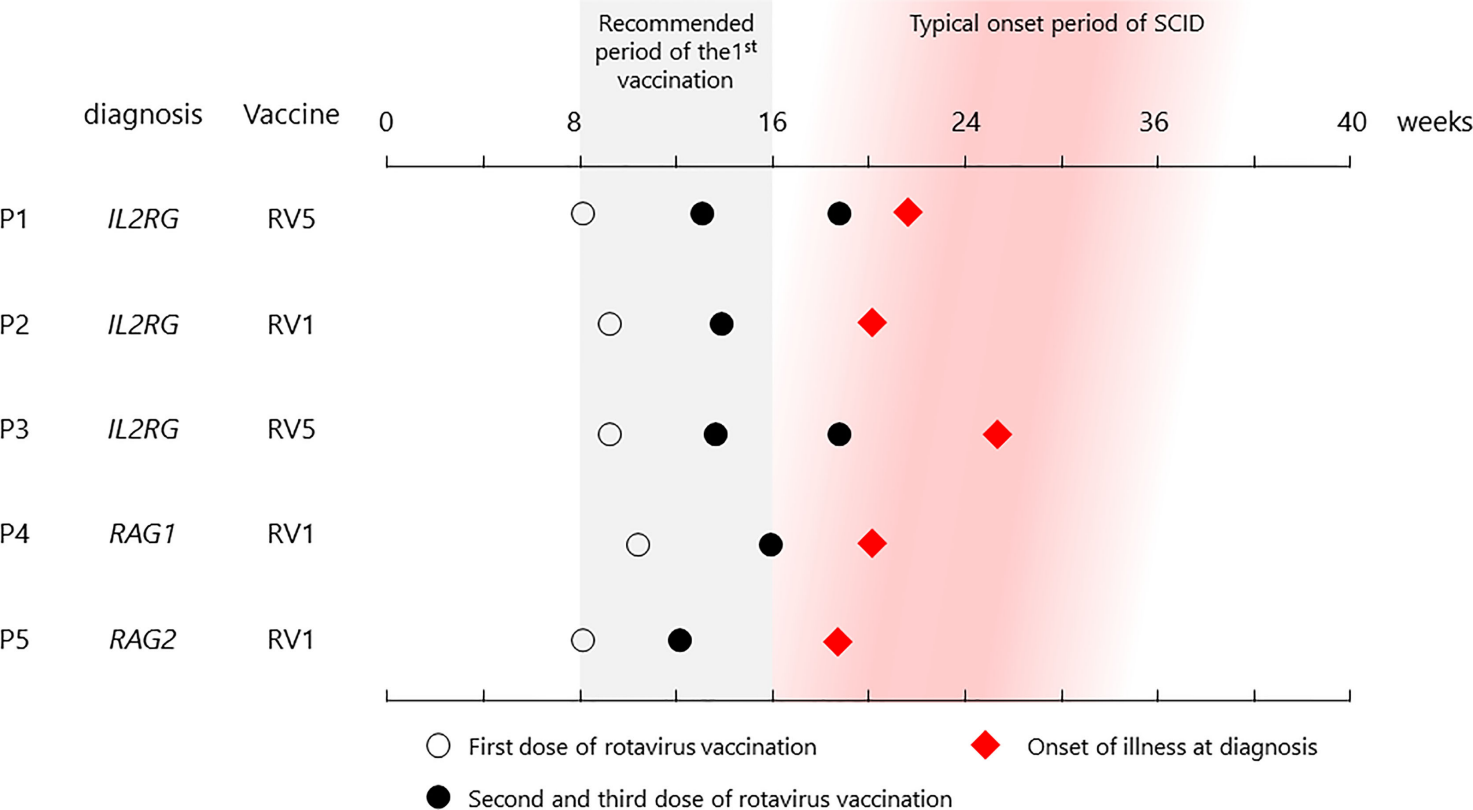
List of vaccination and onset dates in patients. All patients received their first vaccine dose within the recommended period, and each showed symptoms at the typical onset time.

Cases were collected throughout Japan from 2015 to 2020 (**Figure 1** and **Table 1**). The first case (P1) was that of a patient with X-linked SCID, interleukin (IL)-2 receptor gamma (CD132) deficiency, who received the first dose of RV5 at 8 weeks of age (5, 8). He developed respiratory syncytial virus infection and interstitial pneumonia at 5 months of age and was admitted to the hospital. Upon recovery after a 3-week treatment, he had splenomegaly and hypergammaglobulinemia (immunoglobulin G (IgG) > 3,000 mg/dl). Further examination revealed natural killer (NK)-cell deficiency and skewing to memory CD4^+^ T cells, and IEI was suspected. Genetic analysis revealed the causative agent to be the *IL2RG* variant (c.676C>T, p.R226C). However, while preparing for HCT, the patient experienced loose stools, and a rapid test showed that he was rotavirus-positive. Cord blood transplantation (CBT) was performed at 11 months of age at Tokyo Medical and Dental University. The patient presented with loose stools from day 0 to day 11 after CBT. Rotavirus antigen test turned negative on day 28.

**Table 1:** Clinical features of 5 cases.

	P1	P2	P3	P4	P5
Gene	*IL2RG*	*IL2RG*	*IL2RG*	*RAG1*	*RAG2*
Variants	c.676C>T p.R226C	c.202 G>T p.E68*	c.74_75insAC p.T26Rfs	c.2209C>T p.R737C c.2923C>T p.R975W	c.143T>A p.L48Q c.419A>G p.H140R
Vaccine	RV5	RV1	RV5	RV1	RV1
Total dose	3	2	3	2	2
Severity of GI symptoms	Mild	None	Severe	Mild	Mild
Post HCT days when antigen test turned negative	Day 14	Day 32	Day 27	Day 15	Day 38
Weight loss due to GI symptoms	None	None	Severe	None	None
TPN	No	No	No	No	No

RV1, Rotarix^®^; RV5, RotaTeq^®^; GI, gastrointestinal; HCT, hematopoietic cell transplantation; TPN, total parenteral nutrition.

The second case (P2), also with X-SCID, was administered the first dose of RV1 at 9 weeks of age. He developed severe *Pneumocystis* pneumonia (PCP) at 4 months, requiring mechanical ventilation. Flow cytometry revealed NK-cell deficiency with maternal T cells and lack of CD132 on lymphocytes, and the patient was diagnosed with X-SCID with the *IL2RG* variant (c.202 G>T, p.E68*). Although gastrointestinal symptoms were not the main symptoms, the patient tested positive for the rotavirus antigen in the screening test on admission. The patient underwent CBT at 6 months of age (27 weeks 1 day) at the National Center for Child Health and Development. Rotavirus antigen test turned negative 32 days after CBT.

The third case (P3) was that of a patient with X-SCID who was vaccinated with RV5 at 9 weeks of age (4). Three vaccine doses were administered; however, the patient was admitted to the hospital because of diarrhea and weight loss (max −15%) at 5 months. He also had agammaglobulinemia, a lack of T cells and CD132, and was diagnosed with X-SCID (*IL2RG*: c.74_75insAC, p.T26Rfs). The patient underwent CBT at 9 months of age (42 weeks 6 days) at Hokkaido University; however, he remained positive for rotavirus antigen until then. Rotavirus antigen turned negative 27 days after CBT, and steady body weight gain was achieved since then.

The fourth case (P4) was that of a boy with recombination activating gene 1 (RAG1) deficiency, an autosomal recessive SCID, and was vaccinated with RV1 at 10 weeks of age. He was initially admitted to the Kyushu University Hospital at 4 months of age with rhinovirus infection and interstitial pneumonia and was then diagnosed with RAG1 deficiency (*RAG1*: c.2209C>T, p.R737C and c.2923C>T, p.R975W). In addition, he had loose stools and was positive for the rotavirus antigen. He underwent bone marrow transplantation (BMT) from HLA-matched sibling at 6 months of age (26 weeks 0 days). Rotavirus antigen test turned negative 15 days after BMT.

The fifth case (P5) was that of a girl with autosomal recessive SCID, RAG2 deficiency (*RAG2*: c.143T>A, p.L48Q and c.419A>G, p.H140R) who developed PCP at 4 months of age after receiving RV1 (9). During the course of treatment at Nagoya University Hospital for disease management, vomiting and increased gastric remnants were observed, and she was diagnosed as having rotavirus antigen-positive gastroenteritis. Thus, the first CBT was performed at 8 months of age without conditioning treatment because of severe PCP. Although she had mix chimerism, rotavirus antigen test turned negative 38 days after the first CBT. The second CBT with conditioning regimen was performed at 17 months, and she achieved full chimerism and revolved PCP completely.

In all cases, each patient completed the default doses of RV1 or RV5. One case (P2) did not demonstrate GI symptom, and rotavirus antigen was incidentally identified by screening on admission. Two cases (P1 and P4) had minor symptoms with loose stools, and one (P5) had mild symptoms with vomiting. They had no weight loss due to GI symptoms. In contrast, one case (P3) presented with severe diarrhea and weight loss. Total parenteral nutrition was not required in any of them.

### Detection of Vaccine-Derived Rotavirus

**Figure 2:**
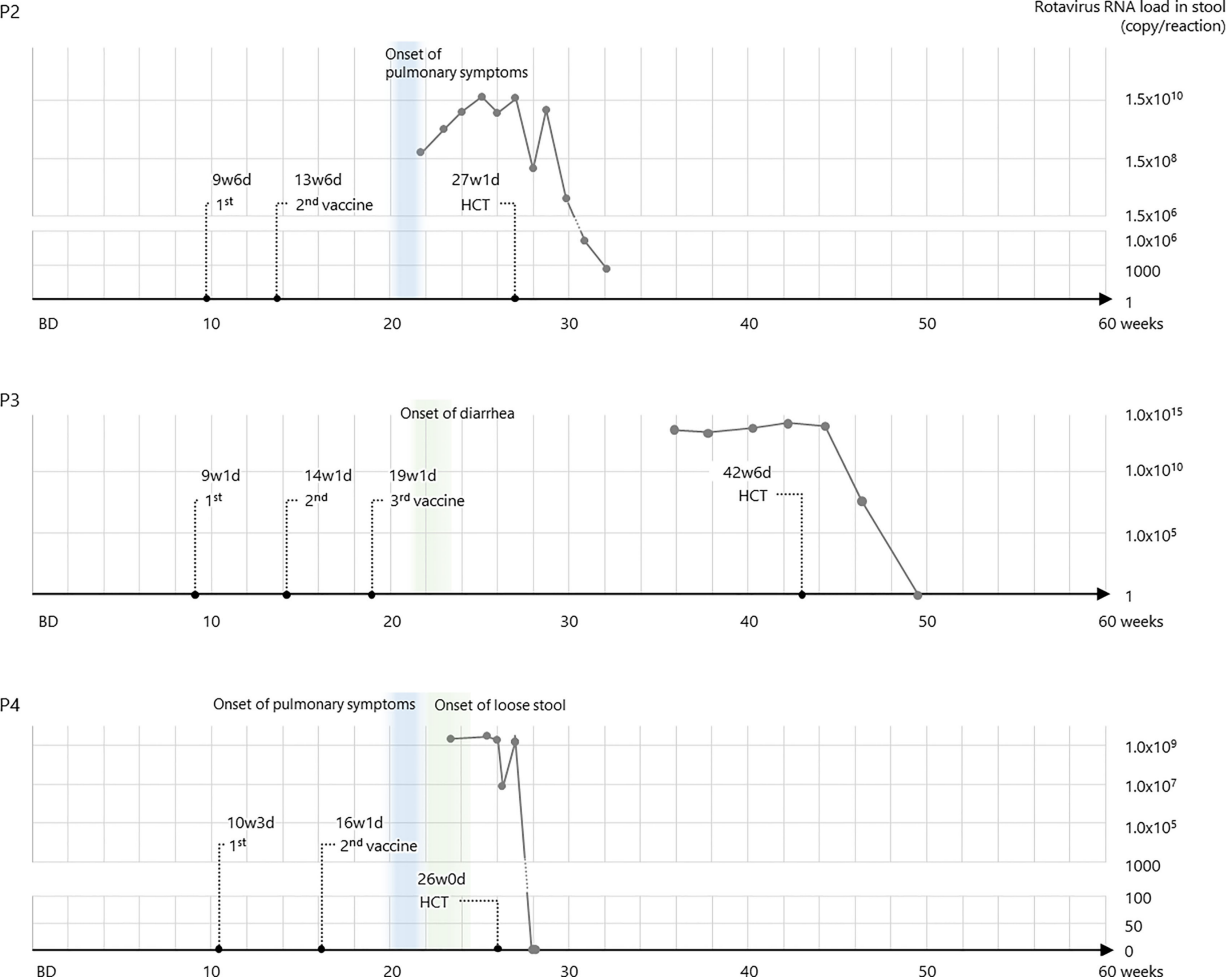
RV5 genotype G1 RNA loads in the stool samples of patients 2, 3, and 4.

Further examination of the rapid test-positive feces revealed that the rotavirus strain from P1 stool was the vaccine-derived strain by Sanger sequence, and those from the others were vaccine strains derived by RT-PCR. Sequential data of viral loads in stools were demonstrated in P2, P3, and P4, and they decreased after HCT (**Figure 2**). In addition, the vaccine strain was detected in the serum of P2 and P4, suggesting that the disease developed into a systemic infection.

## Discussion

Patients with SCID are asymptomatic until 3–4 months of age when maternal IgG levels decrease, making it challenging to diagnose SCID based on physical examination and history alone without knowing family history (1). The recommended age for the first dose of rotavirus vaccine is 8–15 weeks, considering when the infection becomes severe; late administration should be avoided (10). The five SCID cases from Japan suggest that rotaviruses are continuously excreted from the intestinal tract of infected patients, even if they do not show severe gastroenteritis symptoms. Although transmission of the vaccine strain rotavirus is not considered a major problem in healthy infants (11), nosocomial infection thought to be derived from the excreted vaccine strain of rotavirus was observed in P5 (9). Therefore, live rotavirus vaccines should not be administered before diagnosing patients with SCID, even if the disease is not severe. With an annual incidence of approximately 1 in 50,000, SCID is not a rare disease compared with other diseases subjected to mass screening of newborns and given the existence of curative treatments, such as HCT and gene therapy, thereby making it appropriate for screening (12) (unfortunately, gene therapy is not available in Japan). Early diagnosis is necessary to exclude patients with SCID from live rotavirus vaccination targets. T-cell receptor excision circles (TRECs) quantitative PCR assay using neonatal dried blood spots is a useful diagnostic method (13).

Furthermore, a recent review of newborn screening with TRECs showed that the sensitivity of the test for SCID is 100%, demonstrating its usefulness (14). In 2018, TRECs screening for newborns was implemented in all states of the United States, spreading globally, including Europe. However, regional disparities in medical care is a problem because only a few regions in Japan have implemented this system. Assuming that 850,000 babies are born annually in Japan, approximately 15–20 per year will be diagnosed with SCID. Therefore, it is desirable to establish a screening system of TRECs for all newborns in Japan at an early stage.

## Concluding Remark

We described five SCID cases associated with rotavirus-derived infection in Japan with some showing severe gastroenteritis. Therefore, we should introduce TRECs screening for newborns to improve prognosis of SCID in Japan.

## Data Availability Statement

The raw data supporting the conclusions of this article will be made available by the authors, without undue reservation.

## Ethics Statement

The studies involving human participants were reviewed and approved by the ethics boards of the Tokyo Medical and Dental University and Fujita Health University School of Medicine. Written informed consent to participate in this study was provided by the participants’ legal guardian/next of kin.

## Author Contributions

KT and YK wrote the manuscript. YK, HirM, and NM performed genetic analysis. TT, KeI, AI, MY, TaY, HidM, NT, TosM, MK, KE, and MI provided clinical information. SO, KoI, and TomM provided critical discussion. TeY revised the manuscript. HK conceptualized the study and revised the manuscript. All authors contributed to the article and approved the submitted version.

## Funding

This study was partly funded by Takeda Science Foundation.

## Conflict of Interest

The authors declare that the research was conducted in the absence of any commercial or financial relationships that could be construed as a potential conflict of interest.​

## Conflict of Interest

All of the authors declare that they have no relevant conflicts of interest.

## Publisher’s Note

All claims expressed in this article are solely those of the authors and do not necessarily represent those of their affiliated organizations, or those of the publisher, the editors and the reviewers. Any product that may be evaluated in this article, or claim that may be made by its manufacturer, is not guaranteed or endorsed by the publisher.
